# Papillon-Lèfevre syndrome with palmoplantar keratoderma and periodontitis, a rare cause of pyrexia of unknown origin: a case report

**DOI:** 10.1186/s13256-015-0773-7

**Published:** 2015-12-18

**Authors:** Somia Iqtadar, Sami Ullah Mumtaz, Sajid Abaidullah

**Affiliations:** North Medical Ward,Mayo Hospital, King Edward Medical University, Lahore, Pakistan

**Keywords:** Papillon-Lefèvre syndrome, Palmoplantar keratoderma, Periodontitis, Pyogenic liver abscess

## Abstract

**Introduction:**

Papillon-Lefèvre Syndrome is a rare autosomal recessive disorder characterized by diffuse, transgradient palmoplantar keratoderma, destructive periodontitis beginning in childhood, premature loss of primary teeth, and frequent cutaneous and systemic pyogenic infections. Pyogenic liver abscess is an uncommon presentation of the disease present in this case.

**Case presentation:**

A 16-year-old Punjabi, Pakistani boy presented to the outpatient department of a tertiary-care hospital of Lahore with high-grade fever of 2 months duration. He had been treated for a pyogenic liver abscess 2 years back with antibiotics followed by incision and drainage. He had poor orodental hygiene, palmoplantar keratoderma and periodontitis. His parents had history of consanguinity. His brother and two cousins had similar skin lesions and were edentulous. An orthopentogram showed atrophy of the alveolar bone. He was treated with broad-spectrum antibiotics, and antipyretics for systemic infection. Multivitamins, topical steroids, topical keratolytics and emollients were used for his dermatological issues.

**Conclusions:**

Our patient was successfully treated. His fever settled and his skin lesions improved with antibiotics, topical steroids and keratolytics. He was sent home and was asked to return for follow-up on a monthly basis.

## Introduction

Papillon-Lefèvre syndrome (PLS) is a rare autosomal recessive disorder characterized by diffuse, transgradient palmoplantar keratoderma (PPK), destructive periodontitis beginning in childhood, premature loss of primary teeth and frequent cutaneous and systemic pyogenic infections [1]. The syndrome is believed to affect one to four people per million. To date more than 200 cases have been reported worldwide [2]. Without treatment, most of the secondary (permanent) teeth may also be lost. Additional symptoms and findings may include frequent pyogenic skin infections, nail dystrophy and hyperhydrosis [3]. Patients typically have an underlying disease associated with functional or quantitative neutrophil abnormalities and 50 % are immunocompromised [4]. Pyogenic liver abscess is an uncommon presentation of the disease. Patients with PLS seem to be particularly predisposed to develop pyogenic liver abscess [5, 6].

**Fig. 1:**
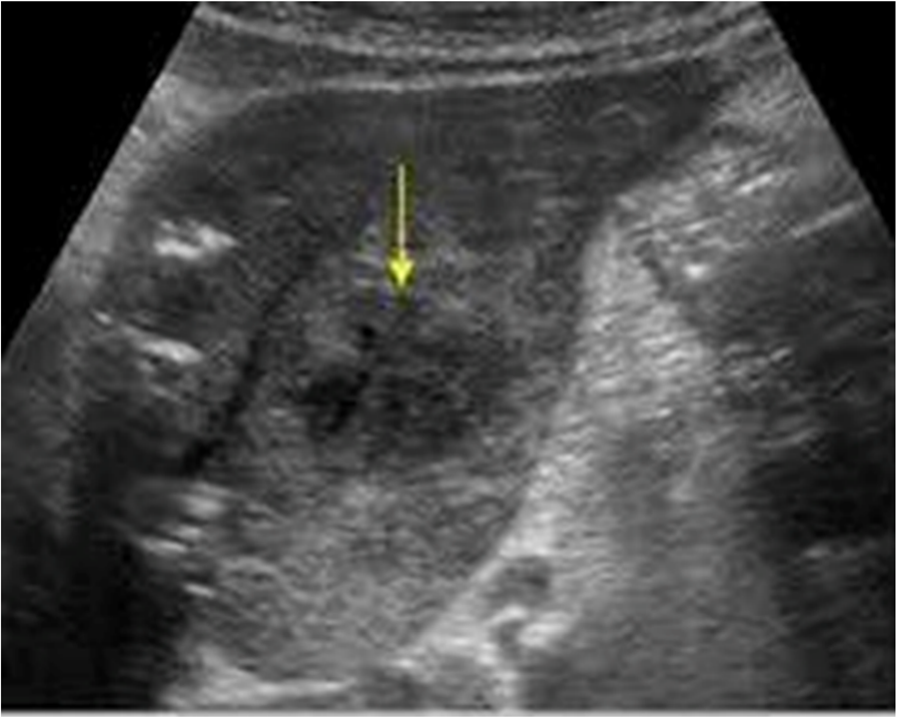
Abdominal ultrasound image of hepatic abscess (old). The arrow is pointing towards hepatic abscess

**Fig. 2:**
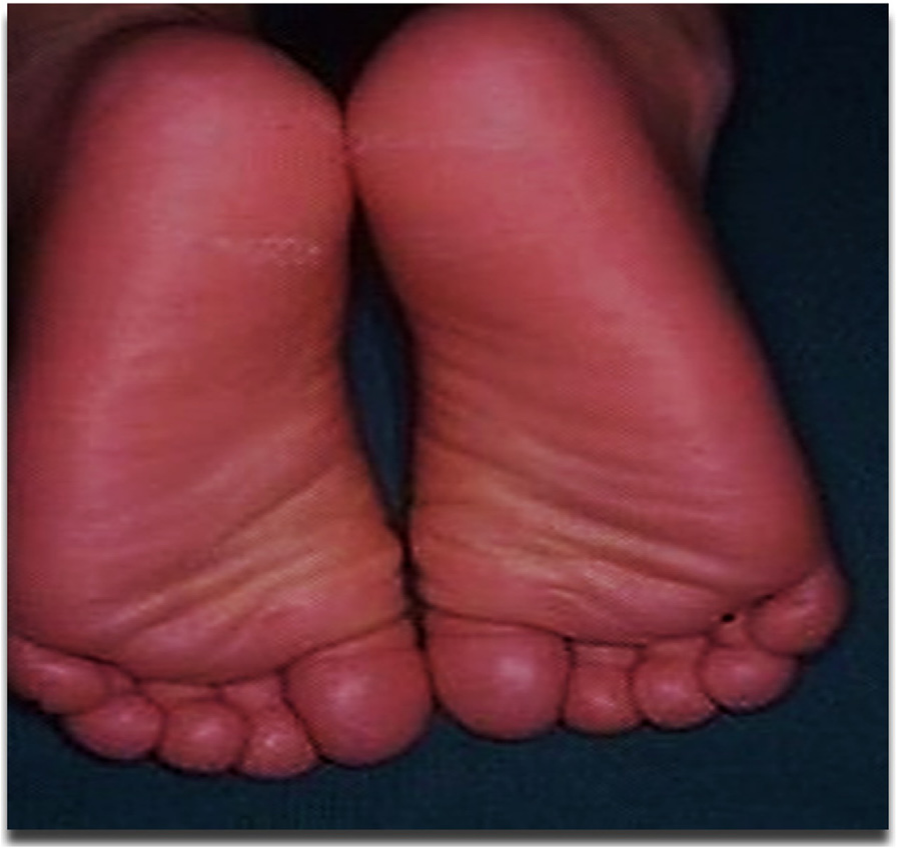
Plantar keratoderma

## Case presentation

A 16-year-old Punjabi, Pakistani boy presented to the outpatient department (OPD) of a tertiary-care hospital of Lahore with high-grade fever of 2 months duration. The fever was gradual in onset, high-grade, continuous and was associated with rigors and chills with peaks in the morning and evening. His fever relieved with antipyretics and cold sponging and there was no aggravating factor. It was associated with persistent productive cough of 2 months duration, with whitish sputum, scanty in amount, about six to seven episodes daily which showed partial response to medication with frequent recurrence. There was also history of anorexia and moderate weight loss. He had been treated for a pyogenic liver abscess 2 years previously with antibiotics followed by incision and drainage (Fig. 1). There was also history of acute gastroenteritis, urinary and respiratory tract infections multiple times within the past year. His parents had history of consanguinity. His brother and two cousins had similar history and were edentulous.

General physical examination showed a young boy of normal height and build with a blood pressure of 120/70 mmHg, pulse rate of 116/minute, regular, temperature of 101 degrees Fahrenheit and respiratory rate of 20/minute. He was pale with poor orodental hygiene. Palmoplantar keratoderma was also noted (Fig. 2).

On examining his gastrointestinal system, spleen was just palpable with no other visceromegaly. Respiratory system examination showed normal vesicular breathing with coarse crepitations in both lung fields. Cardiovascular examination was unremarkable.

His blood profile showed hemoglobin (Hb) of 9.3 mg/dL a total leukocyte count of 11,400/L, and an erythrocyte sedimentation rate (ESR) 68 mm in the first hour. His liver function and alkaline phosphatase test results were slightly raised. His sputum test result for acid-fast bacilli (AFB) was negative on 3 consecutive days. His stool examination, thick and thin blood smears for malarial parasites, typhidot test to detect immunoglobulin (Ig)M and IgG, bone marrow examination and serological test results for hepatitis B surface antigen (HBsAg), anti-hepatitis C virus (HCV), venereal disease research laboratory (VDRL) and human immunodeficiency virus (HIV) were negative. His blood culture showed pseudomonas infection. Chest X-ray showed soft tissue shadows throughout both lung zones. An orthopentogram showed atrophy of the alveolar bone. His medical record showed an abdominal ultrasound performed 2 years ago that showed a hepatic abscess for which he was treated. He had reported a recurrence of abscess 6 months previously (Fig. 1). He was treated with injectable ceftriaxone 2 g per day for 7 days and oral acetoaminophen 4 g per day. A multivitamin tablet containing vitamin C 500 mg, nicotinamide 100 mg, vitamin E 30 IU, calcium pantothenate (pantothenic acid) 20 mg, vitamin B1 (thiamine) 15 mg, vitamin B2 (riboflavin) 15 mg, vitamin B6 (pyridoxine hydrochloride) 20 mg, vitamin B12 12 mcg, folic acid 150 mcg, zinc (equivalent to 100 mg of zinc sulfate) 22.5 mg was also added to the treatment regime in consultation with a dermatologist. He was prescribed topical steroids (betamethasone), topical keratolytics (salicylic acid) and emollients for his skin lesions. Opinion was also taken from the dental surgeon team (the periodontist, pedodontist, and prosthodontist).

Based on history, clinical examination and investigations, a diagnosis of Papillon-Lefèvre syndrome was made.

## Discussion

Papillon-Lefèvre syndrome was first described by Papillon and Lefèvre in 1924. This disease is characterized by diffuse palmoplantar hyperkeratosis and juvenile periodontitis [1, 5]. PPK usually manifests during the first 4 years of life with sharply demarcated hyperkeratosis, more pronounced on the soles of feet and possibly extending to the dorsa of the hands and feet. Erythematous hyperkeratotic plaques may also be present at the elbows, knees, and trunk. The second major feature of PLS is severe periodontitis, which starts at the age of 3 or 4 years and affects both the deciduous and permanent teeth. The teeth erupt normally but are soon lost, and by the age of 14 years, patients are usually edentulous [5].

The underlying cause of periodontitis is not well understood but is now thought to be related to an abnormal immune system and to invading bacteria in the cementum of the teeth. A decreased peripheral CD3, CD4 and defective production of superoxide radicals by polymorphonuclear leukocytes (burst test) has been described in patients with PLS. Defective chemotaxis of polymorphonuclear leukocytes is also a commonly described abnormality. PLS is caused by mutations in the cathepsin C gene on 11q14. However, Pilger *et al*. also reported a late-onset case without this gene [7]. The differential diagnosis of PLS includes Haim-Munk syndrome and Psoriasis. The skin manifestations of PLS are usually treated topically with emollients, keratolytics including salicylic acid and urea. In winter, its worsening may need systemic therapy. Oral retinoids including acitretin, etretinate and isotretinoin have been shown to be effective treatments for the keratoderma seen in PLS. However, Balcı *et al*. recently reported it noncurative [8].

## Conclusions

Our patient was successfully treated with broad-spectrum antibiotics, antipyretics, multivitamins, topical steroids, and topical keratolytics. His fever settled and skin lesions improved. He was sent home and was asked to return for follow-up in the OPD on monthly basis.

## Consent

Written informed consent was obtained from the patient’s next-of-kin for publication of this case report and any accompanying images. A copy of the written consent is available for review by the Editor-in-Chief of this journal.

## Abbreviations

OPD: outpatient department
PLS: Papillon-Lefèvre syndrome
PPK: Palmoplantar keratoderma
